# Enhancing 3-*O*-methylfunicone production by endophytic *Talaromyces pinophilus* J6 through culture optimization and its anti-*Helicobacter pylori* activity

**DOI:** 10.1007/s00203-026-05028-9

**Published:** 2026-07-09

**Authors:** Marcus V. A. Marques, Dalila N. Loose, Crislaine S. Lima, Stéfane M. Q. Santos, Cecília L. S. Pereira, Gabriel S. Ramos, Lorena C. Queiroz, Rita C. R. Gonçalves, Eliane O. Silva

**Affiliations:** 1 Department of Organic Chemistry, Institute of Chemistry, Federal University of Bahia (UFBA), Barão de Jeremoabo 147, Salvador, Bahia 40170-115 Brazil; 2 Department of Pharmaceutical Sciences, Graduate Program in Pharmaceutical Sciences, Center of Health Sciences, Federal University of Espirito Santo, Vitoria, Espirito Santo 29047-105 Brazil

**Keywords:** *Talaromyces pinophilus*, 3-*O*-methylfunicone, Culture optimization, Endophytic fungi, *Helicobacter pylori*, Molecular docking

## Abstract

Endophytic fungi can produce metabolites with potential pharmaceutical applications through biosynthetic pathways strongly influenced by culture conditions. In this study, growth parameters (media composition and cultivation time) were systematically optimized to maximize 3-*O*-methylfunicone (3OMF) production by the endophytic fungus *Talaromyces pinophilus* J6. Chemical profiling by high-resolution mass spectrometry (HRMS) demonstrated that supplementation of potato dextrose agar (PDA) with 45% (w/w) ammonium sulfate and cultivation for 7 days significantly increased the accumulation of 3OMF compared to standard growth conditions. The optimized culture condition enabled the isolation of the target compound in sufficient yield for subsequent biological assays. The compound exhibited anti-*Helicobacter pylori* activity against one reference strain and three clinical isolates, with minimum inhibitory concentrations ranging from 0.165 to 0.660 mM and bactericidal concentrations up to 1.32 mM. In combination assays, 3OMF showed an additive effect with clarithromycin, indicating potential as an adjuvant compound in eradication therapies. Cytotoxicity assays of 3OMF demonstrated selective activity against gastric adenocarcinoma cells, with an IC_50_ of 0.24 mM and selectivity index of 2.37 relative to fibroblasts. Molecular docking suggested favorable interactions between 3OMF and *H. pylori* β-clamp protein, suggesting a possible association with interference in bacterial DNA replication. These findings demonstrate that culture optimization is an efficient biotechnological strategy to improve 3OMF production and support the further investigation of this compound as a scaffold for anti-*H. pylori* applications.

**Graphical abstract:**

**Figure:**
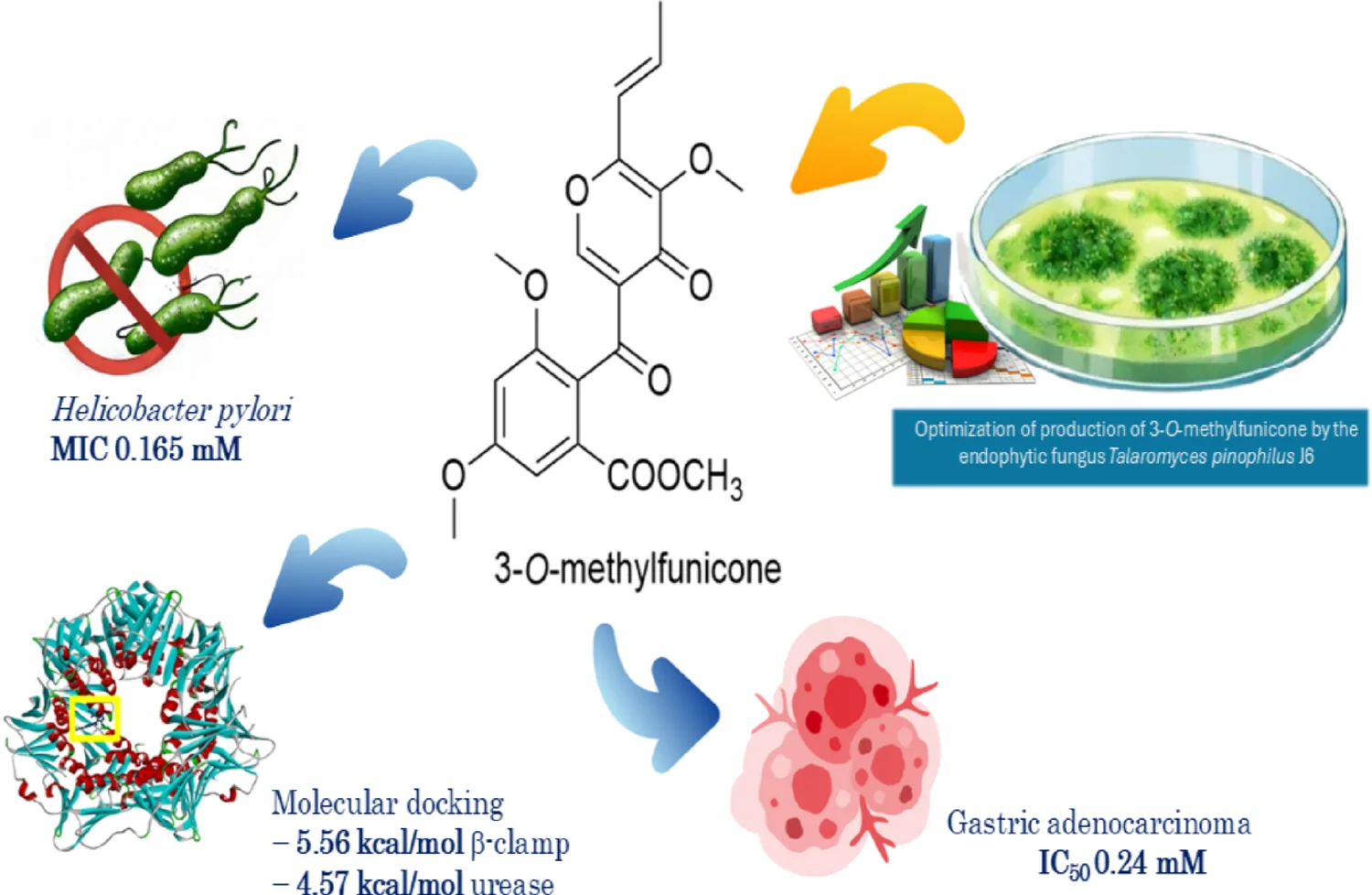

**Supplementary Information:**

The online version contains supplementary material available at https://doi.org/10.1007/s00203-026-05028-9.

## Introduction

*Helicobacter pylori* is a highly prevalent chronic bacterial pathogen that colonizes the human gastric mucosa and persists despite the acidic gastric environment. Clinical outcomes of infection depend on multifactorial interactions among bacterial virulence determinants, host immune responses, and environmental conditions, resulting in variable risks of disease progression, including gastric cancer (Malfertheiner et al. 2023). The bacterium possesses a complex repertoire of proteins that ensure its persistence in the gastric environment while modulating host responses, thereby promoting chronic inflammation (Clyne and Ó Cróinín 2025).

Among these factors, urease is one of the most extensively studied enzymes involved in *H. pylori* pathogenesis. Its activation depends on accessory proteins, particularly the urease maturation protein UreE, which plays a crucial role in delivering nickel ions (Ni²^+^) to apo-urease, the inactive form of the enzyme (Shi et al. 2010). Motility represents another key virulence factor required for successful colonization of the gastric epithelium. This process is tightly regulated by chemotactic signaling mediated by CheY, which is transmitted through the middle domain of the flagellar motor to control bacterial movement (Lam et al. 2010). Beyond virulence and colonization, *H. pylori* persistence also relies on essential cellular processes such as DNA replication. In this context, the β-clamp is a critical component of the replication machinery, ensuring high processivity through the formation of a ring-shaped structure that encircles DNA. This configuration allows continuous activity of DNA polymerase III and prevents its premature dissociation from the DNA template (Pandey et al. 2016). The effectiveness of the current *H. pylori* eradication therapies that combine proton pump inhibitors with antibiotics is declining due to antibiotic resistance, poor patient adherence, and adverse effects (Elbehiry et al. 2023), highlighting the need for novel anti-*H. pylori* agents, particularly from natural sources.

Fungal specialized metabolites are a rich, structurally diverse source of bioactive compounds that have significantly contributed to the development of modern pharmaceuticals. Additionally, multi-omic approaches have greatly accelerated the discovery and characterization of novel fungal natural products (Shankar and Sharma 2022). Endophytic fungi inhabit plant tissues where they establish asymptomatic associations and produce an impressive array of specialized metabolites due to superior biosynthetic capabilities, which may be partially associated with evolutionary adaptation and possible genetic exchange with host plants (Le Cocq et al. 2017). The discovery of new antimicrobial agents from endophytic fungal cultures has often been reported (Ma et al. 2022; Digra and Nonzom 2023), including compounds exhibiting potent anti-*H. pylori* activity (Li et al. 2005).

The biosynthesis of fungal specialized metabolites is highly dynamic and strongly influenced by environmental and nutritional factors. Under laboratory conditions, many biosynthetic gene clusters remain silent and are described as ‘orphan’ and ‘cryptic’, harboring an enormous reservoir of novel bioactive constituents for drug discovery (Zarins-Tutt et al. 2016). Culture-based strategies, such as the One Strain-Many Compounds (OSMAC) approach, have been widely employed to activate or enhance metabolite production by modulating parameters including culture medium composition and cultivation time (Pinedo-Rivilla et al. 2022). These approaches provide an effective means of accessing cryptic or low-abundance metabolites without the need for genetic manipulation.

The fungal *Talaromyces* genus produces a broad array of polyketide metabolites with diverse biological activities (Guo et al. 2016; Lv et al. 2023). Among these metabolites, the funicone derivatives are formed by a γ-pyrone linked to a α-resorcylic acid core and have attracted attention due to their structural features and diverse biological activities (Salvatore et al. 2022). For instance, antifungal activity of 3-*O*-methylfunicone (3OMF) has been extensively reported (De Stefano et al. 1999; Santos et al. 2025). Several *Talaromyces* species, traditionally recognized as soil- and food-associated filamentous fungi, have also been reported as endophytes in diverse plants (Zhang et al. 2024). As endophytes, they adapt their biosynthetic potential to the plant microenvironment, leading to the production of compounds with antifungal, antibacterial, antioxidant, and plant growth-promoting activities (da Silva et al. 2017; Kharkwal et al. 2024). The host-fungus chemical interaction may trigger otherwise silent biosynthetic gene clusters, distinguishing endophytic *Talaromyces* chemistry from that of their free-living counterparts, making them a valuable reservoir of novel bioactive natural products.

Despite 3OMF bioactivity, systematic investigations addressing strategies to improve its production are still scarce. Optimizing culture conditions to enhance 3OMF biosynthesis is therefore essential to obtain sufficient material for biological assays and to enable a more comprehensive assessment of its pharmacological properties. In this study, we investigated the effects of culture media composition and cultivation time on 3OMF production by the endophytic fungus *Talaromyces pinophilus* J6. Liquid chromatography-tandem high-resolution mass spectrometry (UHPLC-HRMS) was employed to identify conditions that maximize 3OMF accumulation. Furthermore, 3OMF was isolated, and its anti-H. *pylori* activity was comprehensively evaluated.

## Materials and methods

### Endophytic fungus isolation and identification

The endophytic fungus employed in the present study was isolated from aerial parts of *Euphorbia umbellata* (Pax) Bruyns (Euphorbiaceae), as previously reported by our research group (Pereira et al. 2026). The host plant was collected from the Atlantic Forest-Cerrado transition area in Bahia State (S 14°54’46.81’, W 40°48’02.33’) and identified by Dr. Nádia Roque (Institute of Biology, Federal University of Bahia). A voucher specimen was deposited in the Alexandre Leal Costa Herbarium (Federal University of Bahia) under the code ALBC 136,527. The study was registered in the Brazilian System for the Management of Genetic Heritage and Associated Traditional Knowledge (SisGen) under code AE18457.

The endophyte strain was identified by DNA sequencing followed by phylogenetic analysis. Genomic DNA was extracted from 7-day-old cultures using mechanical disruption with glass microspheres (425–600 μm diameter; Sigma-Aldrich). PCR amplification was performed using the β-tubulin (benA) gene-specific marker locus with primers Bt2a/Bt2b. PCR reactions were performed in a Thermal Cycler (C1000 Touch) under conditions previously described (Manganyi et al. 2018). Amplification was confirmed by agarose gel electrophoresis and observed under ultraviolet light before purification. Amplicons were then purified using the GFX PCR DNA and Gel Band Purification Kit (GE Healthcare) and sequenced on an ABI 3500XL Genetic Analyzer (Applied Biosystems). Consensus sequences were manually edited and assembled using BioEdit (ver. 7.2.6), then compared against sequences in the NCBI database with the Basic Local Alignment Search Tool (BLAST; https://blast.ncbi.nlm.nih.gov/). Fungal identification was assigned based on the highest sequence similarity with reference strains deposited in GenBank (https://www.ncbi.nlm.nih.gov/genbank/).

For phylogenetic analysis, sequences were aligned with ClustalX (Thompson 1997) and analyzed in MEGA (ver. 11.0) (Tamura et al. 2021). A phylogenetic tree was reconstructed using the Neighbor-Joining method with the Kimura two-parameter (K2P) model (Kimura 1980) which was used to calculate evolutionary distance matrices. Branch support was evaluated with 1,000 bootstrap replicates. The obtained sequence was deposited in GenBank under accession number PQ963936.

### Endophytic fungi cultures, metabolic extraction, and quantification of 3OMF

To evaluate the accumulation of the specialized metabolite 3OMF in crude extracts of strain J6, the fungus was cultivated in three different culture media over 7 days. The endophytic fungal strain J6 was grown in Petri dishes containing 20 mL of Potato Dextrose Agar (PDA), PDA modified with 45% (w/w) sodium tartrate dihydrate (PDA_ST), or PDA modified with 45% (w/w) ammonium sulfate (PDA_AS). All cultures were prepared in triplicate and incubated simultaneously in a Biochemical Oxygen Demand (BOD) chamber at 28 °C.

Samples were collected daily throughout the cultivation period. Crude extracts were obtained by adding 40 mL of ethyl acetate directly to each culture plate, including both the culture medium and fungal mycelium. The mixtures were sonicated for 20 min, filtered, and the organic phase was concentrated under reduced pressure at 40 °C until dryness. Blank extractions were performed in parallel under identical conditions using uninoculated PDA, PDA_ST, and PDA_AS media.

Absolute quantification of 3OMF was performed using an external calibration curve (Figure S2) constructed with a purified 3OMF standard. The calibration curve was generated by correlating chromatographic peak area with known concentrations of 3OMF and was used to determine the absolute concentration of 3OMF in each extract. Quantification was performed using three independent biological replicates per cultivation day and culture condition. The results are reported as mean concentration ± standard deviation.

### UHPLC-HRMS analysis

All crude extracts from endophyte cultures were analyzed by ultrahigh-performance liquid chromatography coupled with high-resolution mass spectrometry (UHPLC-HRMS). MS and MS2 data were acquired using data-independent acquisition (DIA) in both positive and negative ion modes. The UHPLC-HRMS apparatus (Thermo Fisher Scientific) consisted of an electrospray ionization (ESI) source and an Orbitrap analyzer. The flow rate was 400 µL/min, and the gradient elution system was 5 to 100% methanol (HPLC grade Tedia) in water for over 35 min. The C18 column (ACE 150 mm×4.6 mm×3 μm) temperature was set to 30 °C. The following parameters were used: a scanning range of 120–1200 *m*/*z* for full MS, ESI MS resolution of 70.000 with lock mass, a microbeam of 1, and a maximum injection time of 250 ms. The parameters of the ESI ionization source were as follows: gas flow rate of 30 L/min; auxiliary gas flow rate of 10 L/min; positive voltage spray mode of 3.6 kV; negative voltage spray mode of 3.2 kV; and Slens level of 55. Nitrogen gas was used as a nebulizer in the collision cell.

The mass spectra were processed using Xcalibur software (Thermo Fisher Scientific). All base peak chromatograms (BPC) data were inspected to verify the detection of 3OMF. MS2 spectra corresponding to 3OMF were then extracted and analyzed, and the fragmentation patterns were proposed based on diagnostic product ions and neutral losses.

### Isolation and chemical identification of 3OMF

The endophytic fungus J6 was cultivated in 100 Petri dishes (90 mm) containing PDA medium supplemented with ammonium sulfate (45% w/w) and incubated in a BOD chamber for 7 days at 28 °C. The ethyl acetate extract was dried over anhydrous sodium sulfate and concentrated under reduced pressure at 40 °C. The purification process was carried out on a chromatographic column (40 × 1.5 cm) containing silica gel 60 A (Sigma-Aldrich). The mobile phase consisted of hexane (Synth), ethyl acetate (Synth), and methanol (Synth) in gradient elution. A total of 10 fractions were collected. The ninth fraction was subjected to further purification by solid-phase extraction (SPE) using a Visiprep-SPE (Supelco) system with a reverse-phase column. The sample was added to C_18_ cartridge, which was eluted with increasing portions of methanol in water. Finally, the polyketide 3OMF was achieved as pure compound.

One- and two-dimensional NMR spectra were recorded at 500 MHz and 125 MHz for ^1^H and ^13^C, respectively, using a DRX 500 spectrometer (Bruker). Chemical shifts (*δ*) were referenced to the residual deuterated methanol (CD_3_OD) peak at *δ*_H_ 3.31 for ^1^H and *δ*_C_ 49.00 for ^13^C.

(*E*)−3-methoxy-2-propenyl-5-(2′-carbomethoxy-4′−6′-dimethoxybenzoyl)−4-pyrone or 3-*O*-methylfunicone (3OMF): white powder; ^1^H-NMR (500 MHz, CD_3_OD): *δ* 8.48 (1H, *s*, H-9); 7.07 (1H, *d*, *J* = 2.0 Hz, H-6); 6.81 (1H, *d*, *J* = 2.0 Hz, H-4); 6.74 (1H, *d*, *J* = 6.7 Hz, H-15); 6.61 (1H, *dd*, *J* = 15.8 and 1.7 Hz, H-14), 3.88 (3H, *s*, 5-OCH_3_), 3.78 (3H, *s*, 7-COOCH_3_), 3.76 (3H, *s*, 12-OCH_3_), 3.75 (3H, *s*, 3-OCH_3_), 1.97 (3H, *dd*, *J* = 6.7 and 1.7 Hz, H-16); ^13^C-NMR (125 MHz, CD_3_OD): *δ* 192.8 (C-1), 174.3 (C-11), 168.0 (7-COO), 163.1 (C-5), 161.5 (C-9), 159.5 (C-3), 156.8 (C-13), 145.2 (C-12), 137.4 (C-15), 132.0 (C-7), 128.0 (C-10), 126.0 (C-2), 119.3 (C-14), 107.3 (C-6), 103.6 (C-4), 61.1 (12-OCH_3_), 56.7 (3-OCH_3_), 56.3 (5-OCH_3_), 52.9 (7-COOCH_3_), 19.0 (C-16). HRMS *m*/*z* 389.1244 ([M + H]^+^; (C_20_H_20_O_8_)H^+^; calc. 389.1231; error 3.3 ppm).

### Combined *in vitro* and *in silico* analysis of anti-*Helicobacter pylori* activity

#### Antimicrobial assay

Anti-*H. pylori* activity was expressed by the Minimal Inhibitory Concentration (MIC) and the Minimal Bactericidal Concentration (MBC), according to the broth microdilution technique proposed by the Clinical and Laboratory Standards Institute (CLSI 2015). Assays were performed with one commercial *H. pylori* strain ATCC 43504 and three clinical strains submitted to GenBank under the following codes: PX256792, PX256793, and PX256794, approved by ethics committee for research involving human subjects CAAE 9877223.2.0000.5060; 6.114.766 - CEP/UFES. Clinical strains were isolated from an endoscopic gastric biopsy. Microscopic analysis revealed the presence of flagellated Gram-negative bacilli, and bacterial identity was confirmed by 16 S rRNA partial sequencing, followed by BLAST comparative analyses with the NCBI database. The three clinical strains were positive for both cagA and vacA genotypes (PX256792 – s0m1, PX256793 – s2m2, and PX256794 – s1m2) and exhibited resistance to amoxicillin while remaining susceptible to clarithromycin and metronidazole (unpublished data) according to the BrCAST–EUCAST clinical breakpoints valid in 2026 (BrCAST, 2026). The reference strain *H. pylori* ATCC 43504 exhibited resistance to metronidazole and susceptibility to clarithromycin and amoxicillin and was also positive for both cagA and vacA genotypes (ATCC, 2026).

For assay, all *H. pylori* strains were cultivated in Columbia Blood Agar (CBA, Newprov) plates and incubated at 37 °C under controlled microaerophilic conditions using a CO₂ incubator maintained at 10% CO₂. Atmospheric conditions were continuously regulated by the incubator’s internal sensor. After 3 to 5 days of growing, colonies were collected and suspended in Brucella broth (HiMedia) with 10% of Fetal Calf Serum (FCS, Vitrocell) at 10^6^ colony-forming units per milliliter (CFU/mL), equivalent to a 1:20 dilution of 0.5 McFarland bacterial suspension. A 3OMF serial dilution was prepared using the same medium at a concentration range of 82.4 to 2636.7 µM. 100 µL of bacterial suspension and 100 µL of each 3OMF dilution were added to each well of a 96-well plate. The plate was homogenized, read at λ 620 nm, and incubated at 37 °C. After 72 h, the plate was again homogenized and read at the same wavelength. MIC was defined as the lowest concentration that reduced absorbance by 90%. MBC was defined as the lowest concentration that inhibited agar colony growth after 72 h of incubation in CBA. Assays were performed in triplicate. Amoxicillin (Sigma-Aldrich), metronidazole (Sigma-Aldrich), and clarithromycin (Sigma-Aldrich) were used as the standard drugs (positive controls). Controls composed of bacteria without 3OMF (growth control) and pure medium (sterility control) were also performed.

Bacterial cultivation and MIC/MBC assays were performed at 37 °C under controlled microaerophilic conditions using a CO₂ incubator maintained at 10% CO₂. Atmospheric conditions were continuously regulated by the incubator’s internal sensor, and the equipment was periodically calibrated according to institutional laboratory procedures.

#### *H. pylori* urease inhibition assay

Urease inhibition was determined by measuring the ammonia produced by urease-catalyzed hydrolysis (Tanaka et al. 2004; Okyay and Rodrigues 2013).

A bacterial suspension was prepared from 3- to 5-day-old colonies in phosphate-saline buffer pH 6.8. In a 96-well plate, 25 µL of bacterial suspension OD_620_ of 0.50) and 25 µL of 3OMF (32–1024 µg/mL) were incubated for 3 h at 37 °C/10% CO_2_. Then, 50 µL of 100 mM urea solution containing 0.002% of phenol red. Absorbance was read at 540 nm at time zero and after 40 min of reaction, to determine the percentage of inhibition. Acetohydroxamic acid (AHA, Sigma-Aldrich) was used as a standard drug, and phosphate-saline buffer as a reaction control.

#### Synergism assay with standard antibiotics

A checkerboard assay was used to assess the synergistic effect of 3OMF with three standard antibiotics: clarithromycin, amoxicillin, and metronidazole. To do so, different concentrations of 3OMF (MIC, 1/2 MIC, 1/4 MIC, and 1/8 MIC) were combined with different concentrations (MIC, 1/2 MIC, 1/4 MIC, and 1/8 MIC) of each antibiotic, following the same principles of the broth dilution method (Sect. 2.5.1). Treatment interactions are calculated using the fractionated inhibitory concentration index (FICI), which is the sum of the fractionated inhibitory concentrations (FICs) for each sample. FIC is expressed as the ratio between the combined MIC and the single drug MIC. According to FICI values, the effect was classified as synergic (FICI ≤ 0.5), additive (0.5 < FICI ≤ 1.0), indifferent (1.0 < FICI ≤ 4.0), or antagonic (FICI > 4.0) (Krzyżek et al. 2020).

#### Cytotoxicity assay

Gastric adenocarcinoma cells (AGS CRL – 1739) and fibroblast (ATCC NCTN clone 929 CCL-1) were received from “Banco de Células do Rio de Janeiro” (BCRJ Cod. 0311) and cultivated according to the supplier’s recommendations. Cytotoxicity was determined by MTT-assay (3-(4,5-dimethylthiazol-2-yl)−2,5-diphenyltetrazolium bromide). Cells were seeded at a 96-well plate at 1.3 × 10^4^ cells per well and incubated until confluence. Then, they were treated with different concentrations of 3OMF (64 to 2060 µM) and incubated for 48 h. After incubation, the supernatant was removed, and 100 µL of 1 mg/mL MTT solution was added for 2 h. The supernatant was again discarded, and formazan crystals were solubilized in DMSO for absorbance reading at 540/620 nm (Mosmann 1983). Assays were performed in triplicate, using cisplatin as the standard drug and cells in culture medium without 3OMF as the growth control. Results were expressed by median inhibitory concentration (IC_50_), and fibroblast cells were used to calculate the selectivity index (SI).

#### Crystallographic complexes and molecular docking

In order to cover different classes of proteins expressed by *H. pylori*, targets related to DNA replication, motility, chemotaxis, and nitrogen metabolism were preselected. Subsequently, the crystallographic complexes of the proteins were obtained from the Protein Data Bank (PDB), from which the following structures were selected: flagellar export chaperone FliS (31QC), middle domain of the flagellar motor switch protein FliM (4GC8), replicative helicase DnaB (4ZC0), β-clamp elongation factor crystallized with a DNA ligase peptide (5FRQ), response regulator CheY1 crystallized with Mg²⁺ and BeF₃⁻ (3H1E), glutamine synthetase (5ZLI), urease maturation protein UreE crystallized with Ni²⁺ (3NY0).

The three-dimensional structures of the ligands were optimized using the WebMo platform (accessible at https://www.webmo.net/), with MOPAC, employing geometric optimization. The most stable conformers were selected for docking against the target proteins. 3OMF structure was obtained from PubChem and optimized with MMFF94 force field at pH 7.4 using OpenBabel (O’Boyle et al. 2011). Molecular docking calculations were performed by AutoDock Vina (Eberhardt et al. 2021; Trott and Olson 2010). Crystallized structures associated with the target proteins, including water molecules, were removed, and the macromolecules were protonated at physiological pH. Grid boxes were set at 20 Å and centered at each protein’s active site. Molecular docking protocol validation was performed by redocking using the crystallographic ligands associated with each target. Validation was considered acceptable for RMSD (Root Mean Square Deviation) values ≤ 2.0 Å. Representative redocking poses and corresponding RMSD values are presented in the Supplementary Material (Figure S8).

To predict the interaction between ligands and the 3OMF, the AutoDock 4 software was used. In the program, crystallized structures associated with the target proteins, including water molecules, were removed. Subsequently, polar hydrogen atoms were added to both the macromolecule and the ligand, along with Kollman charges. The delimitation of the active site of each protein was carried out based on coordinates extracted from reference ligands using the Discovery Studio program. Finally, the Lamarckian Genetic Algorithm (LGA) was employed.

For proteins that had crystallized ligands, redocking was performed. The preparation of the protein and its ligands, as well as the calculations, followed the same methodology used in molecular docking.

### Statistical analysis

The assays were performed in triplicate and repeated at least three independent times, the results being represented by mean of triplicate ± standard deviation (SD). Statistical analysis was performed using GraphPad Prism (GraphPad Software, San Diego, CA, EUA). Non-linear regression was used for IC_50_ values calculation. MICs were compared by two-way ANOVA followed by Dunnet´s *post-hoc* test, with statistical significance set at *p* < 0.05.

## Results

### Optimizing production of 3OMF by the endophytic fungus

**Fig. 1:**
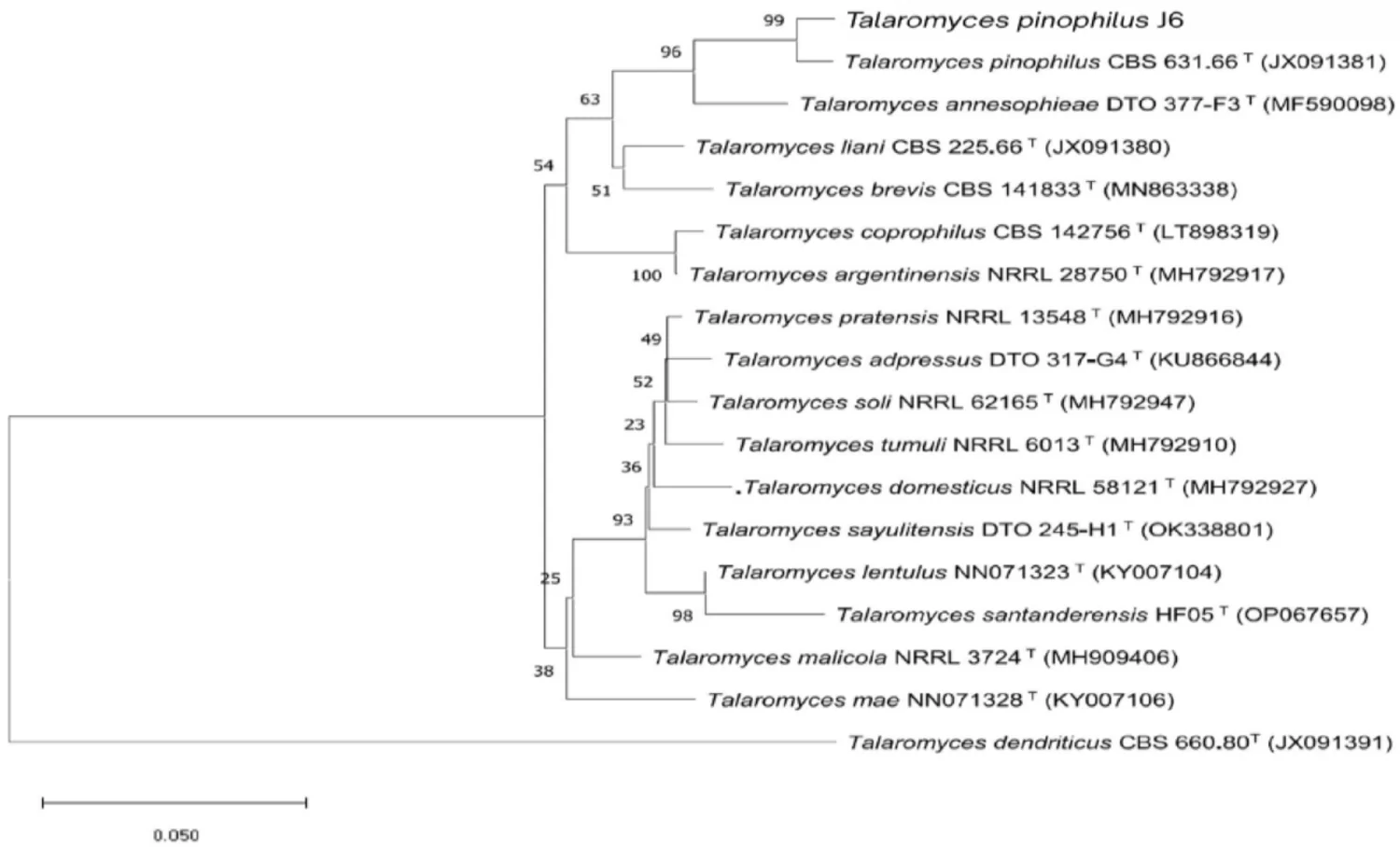
Phylogenetic position based on the BenA sequence of *Talaromyces pinophilus* J6. The phylogenetic tree was constructed using the Neighbor-Joining method in MEGA 6. Bootstrap analysis was performed with 1,000 replications, and the results are indicated at the nodes. The scale bars represent 0.050 substitutions per site

The endophyte strain J6 was isolated from the aerial parts of *Euphorbia umbellata* as previously reported (Pereira et al. 2026). Molecular identification using the benA gene sequence assigned strain J6 to the genus *Talaromyces*, as depicted in the phylogenetic tree shown in Fig. 1. The analysis showed that J6 clusters closely with *Talaromyces pinophilus*, forming a strongly supported clade with *T. pinophilus* CBS 631.66 (JX091381), supported by a bootstrap value of 99%.

*T. pinophilus* J6 was grown for 7 days in Petri dishes containing different culture media: PDA, PDA supplemented with 45% (w/w) sodium tartrate dihydrate (PDA_ST) or ammonium sulfate (PDA_AS). Despite the osmotic stress imposed by the saline medium, J6 demonstrated adequate growth under both conditions (PDA and PDA_AS), indicating its ability to tolerate elevated salt concentrations. Representative cultures after 7 days of incubation under supplemented and non-supplemented conditions are shown in Figure S1 (Supplementary Material).

**Fig. 2:**
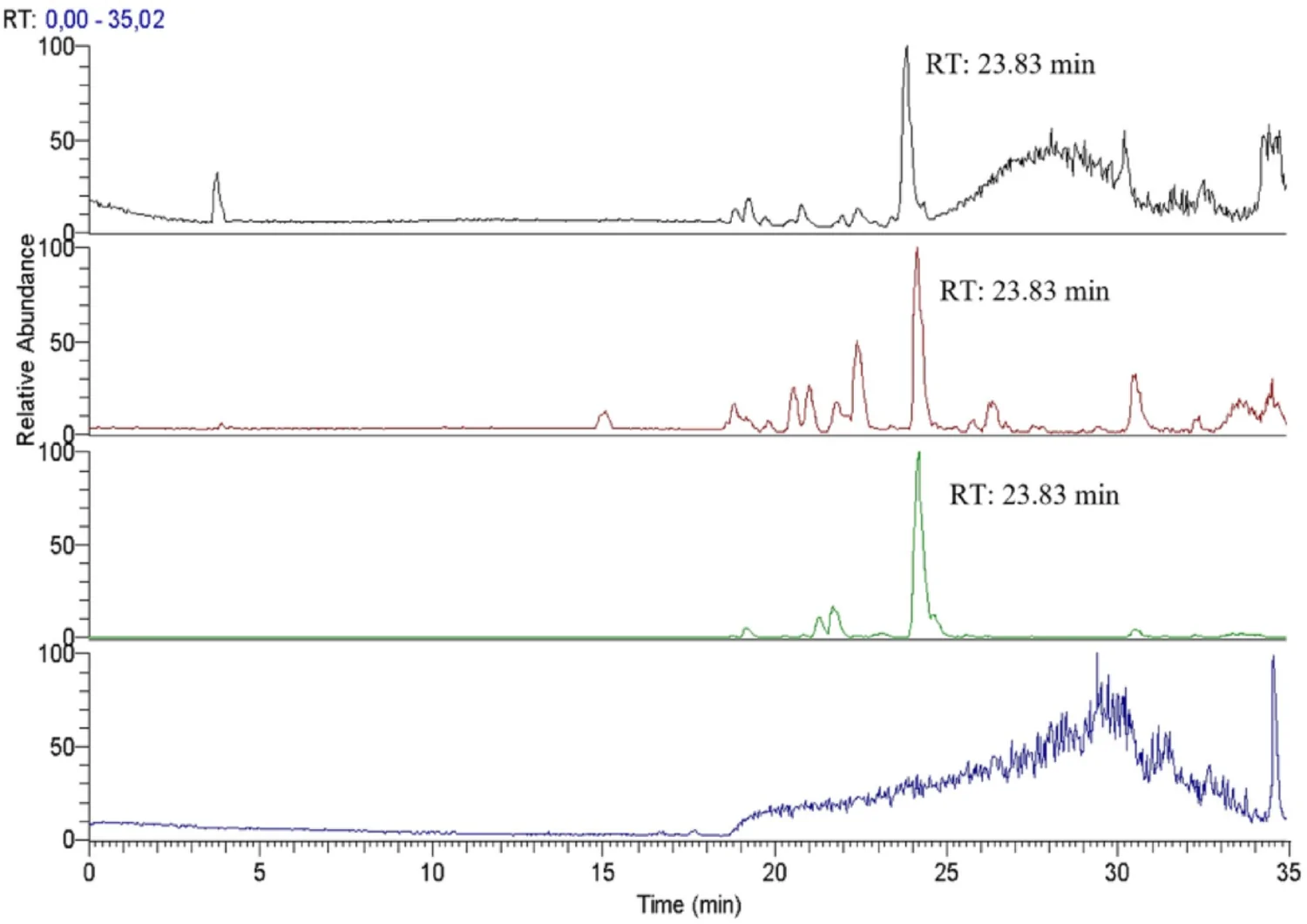
Base peak chromatograms (BPC) of the *Talaromyces pinophilus* J6 extracts obtained from cultures grown in PDA (black), PDA_ST (red), and PDA_AS (green) for 7 days. The blue BPC corresponds to the blank (PDA extract without fungus). The peak at retention time (RT) 23.83 min corresponds to 3OMF (colour figure online)

Ethyl acetate extracts from all cultures were analyzed by UHPLC/ESI-Q-Orbitrap to monitor the biosynthesis of specialized metabolites. Base peak chromatograms (BPCs) (Fig. 2) provide an overview of the chemical complexity of the samples. The positive ionization mode yielded higher signal intensity and was therefore selected for comparison of the chemical profiles.

Figure 2 shows that the base peak chromatograms (BPCs) of the ethyl acetate extracts obtained from *T. pinophilus* J6 cultivated in PDA (black), PDA_ST (red), and PDA_AS (green) media displayed a predominant signal at a retention time of 23.83 min. Extraction and quantitative analysis of the corresponding chromatographic peak revealed values of 1.64E10, 3.44E10, and 3.19E11 for PDA, PDA_ST, and PDA_AS, respectively.

**Fig. 3:**
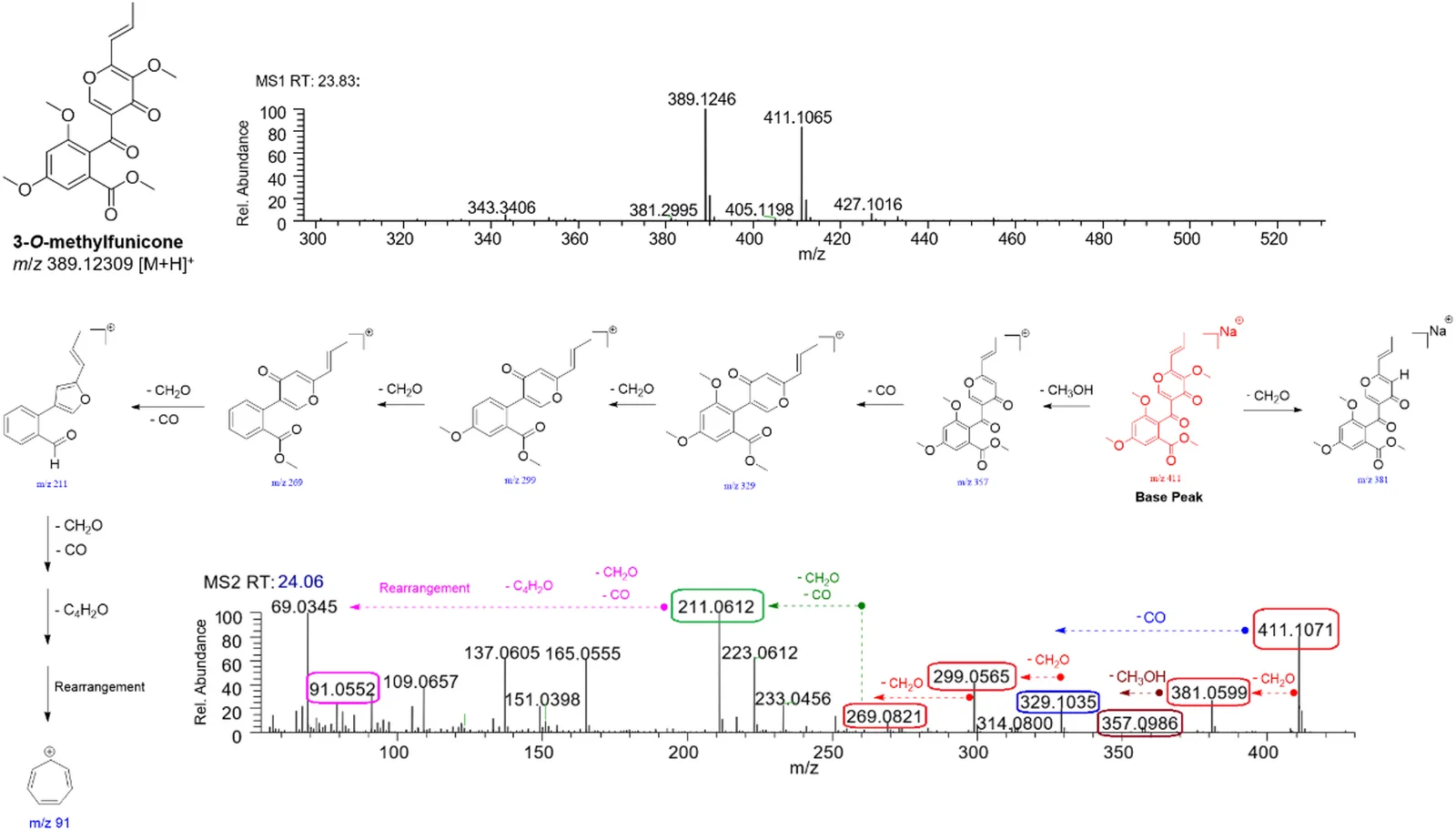
MS1 and MS2 spectra of 3-*O*-methylfunicone (3OMF) and proposed fragmentation pathway

MS1 and MS2 spectra were extracted from the peak at RT 23.83 min to enable the preliminary identification of the main natural product detected at this retention time. The MS1 spectrum (Fig. 3) showed two peaks at *m*/*z* 389.1246 and 411.1065, which are consistent with the protonated molecular ion [M + H]⁺ and the sodium adduct [M + Na]⁺, respectively, of the molecular formula C_20_H_20_O_8_. Subsequently, manual curation of the MS2 spectrum (Fig. 3) was performed to improve the reliability of the annotation. For this purpose, a fragmentation pathway was proposed based on the observed product ions. (Fig. 3). Initial CH_2_O loss from *m*/*z* 411 generated the ion at *m*/*z* 381. On the other hand, neutral CH_3_OH loss from *m*/*z* 411 generated *m*/*z* 357, which was transformed into ions at *m*/*z* 329, 299, 269, 211, and 91 by sequential losses of CO, CH_2_O, and C_2_H_2_. Therefore, the main specialized metabolite detected in crude extracts was putatively identified as 3OMF.

**Graph 1:**
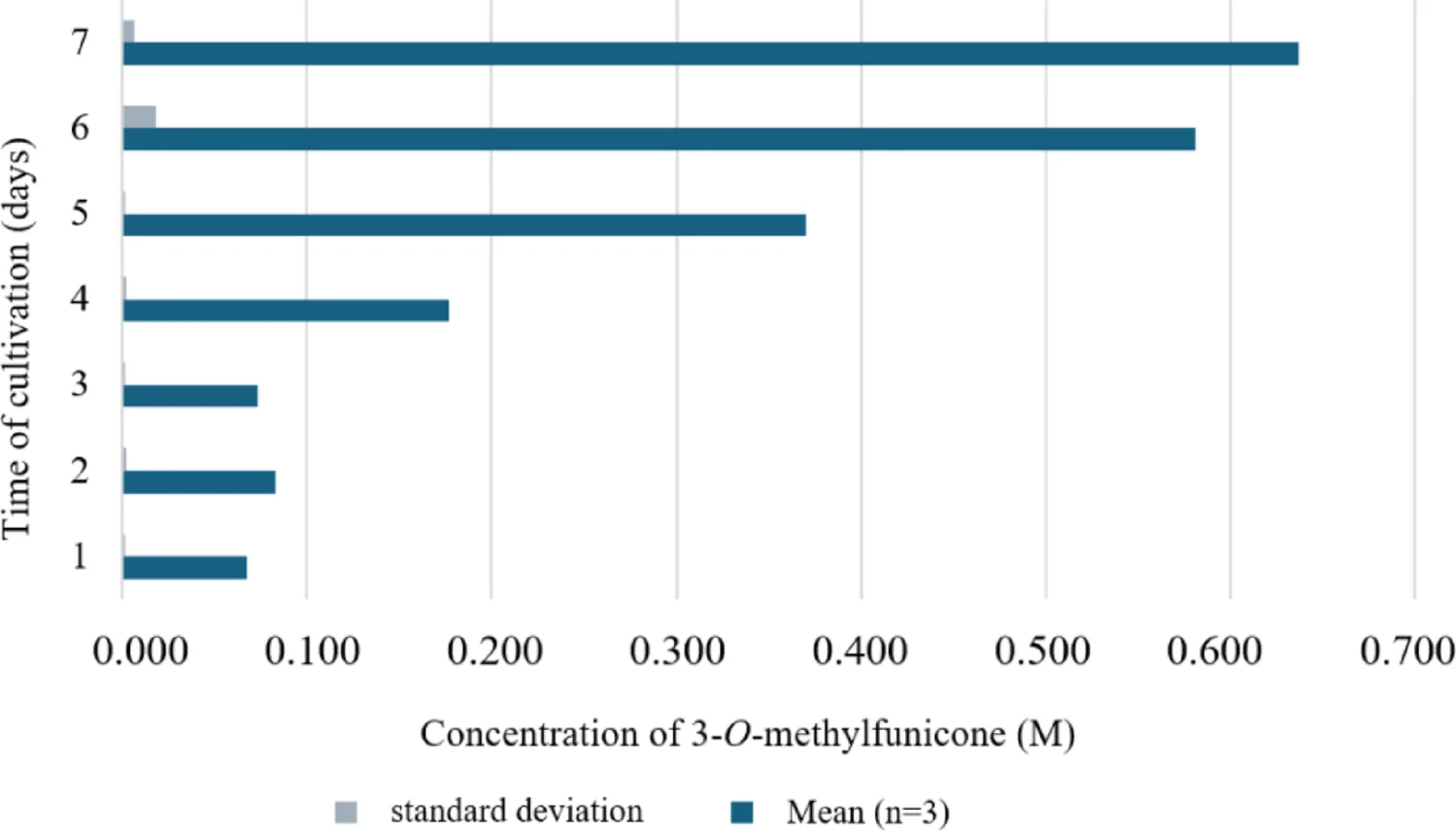
Time-course production of 3OMF during solid-state fermentation. Absolute concentrations of 3-*O*-methylfunicone (M) calculated from the calibration curve presented in Figure S2. Data are expressed as mean ± standard deviation of three independent replicates for each cultivation time point

After MS spectral analysis and confirmation that biosynthesis of the target compound 3OMF was highest in PDA_AS, the optimal cultivation time was subsequently evaluated in this medium. For this purpose, *T. pinophilus* J6 was grown in PDA_AS for seven days, and samples were collected daily to quantify 3OMF accumulation. Time-course monitoring revealed that 3OMF production started on the first day of cultivation and progressively increased, reaching its maximum level on the seventh day (Graph 1).

Finally, scale-up cultivation was performed under optimized conditions to obtain enough 3OMF for subsequent biological assays. The endophytic fungus J6 was cultivated in 100 Petri dishes containing PDA_AS for seven days, yielding 1300 mg of dried ethyl acetate extract. Chromatographic fractionation enabled the isolation of pure 3OMF, obtained in a yield of 65 mg (5% relative to the crude extract).

Analysis of the 1D and 2D NMR data (Figures S3-S6 in Supplementary Material) analysis allowed the unequivocal confirmation of the chemical structure of the 3OMF (De Stefano et al. 1999). Briefly, the singlets at *δ*_H_ 3.88, 3.78, 3.76, and 3.75 were attributed, respectively, to methoxy groups attached at positions 5, 7, 12, and 13. The two ethylenic hydrogens (*δ*_H_ 6.61 and 6.74, at C-14 and C-15, respectively) were in *E* configuration because of the mutual coupling constant (*J* 15.8 Hz). Moreover, the methyl group at *δ*_H_ 1.97 (C-16), was attached at position C-15 based on its correlations with C-14 (*δ*_C_ 119.3) and C-15 (*δ*_C_ 137.4), suggesting a propenyl side chain. The aromatic H-6 and H-4 (*δ*_H_ 7.07 and 6.81, respectively) had a meta coupling constant (*J* 2.2 Hz). Signals at *δ*_C_ 174.3 (C-11), 161.5 (C-9), 156.8 (C-13), 145.2 (C-12), and 128.0 (C-10) in ^13^C NMR, along with the 2D correlations with methine hydrogen *δ*_H_ 8.48 (position 9), were in good agreement for a *γ*-pyrone nucleus.

### *In vitro* and *in silico* anti-*Helicobacter pylori* assays

#### *H. pylori* inhibition and cytotoxicity of 3OMF

The *in vitro* antibacterial activity of 3OMF was evaluated using the microplate microdilution method. Activity was expressed as the Minimum Inhibitory Concentration (MIC), defined as the lowest concentration capable of inhibiting *H. pylori* growth, and the Minimum Bactericidal Concentration (MBC), defined as the lowest concentration that prevented colony formation on agar after 72 h of incubation. The assays were performed against one reference *H. pylori* strain (ATCC 43504) and three clinical isolates (PX256792, PX256793, and PX256794). Amoxicillin, metronidazole, and clarithromycin were used as positive controls.

The results (Table 1 and Figure S5) showed that 3OMF exhibited MIC values of 0.165 mM, 0.660 mM, 0.330 mM, and 0.660 mM against *H. pylori* ATCC 43,504, PX256792, PX256793, and PX256794, respectively. For MBC determination, corresponding values of 0.330 mM, 0.660 mM, 0.330 mM, and 1.32 mM were observed against *H. pylori* ATCC 43,504, PX256792, PX256793, and PX256794, respectively. 3OMF exhibited greater antibacterial activity than metronidazole across all tested strains, as indicated by its markedly lower MIC values.

Statistical analysis of the bacterial growth inhibition data demonstrated a concentration-dependent effect of 3OMF against *H. pylori* strains (Figure S5). Two-way ANOVA followed by Dunnett’s post-hoc test revealed significant differences in growth inhibition at specific concentrations compared with the untreated control (*p* < 0.05). These findings support the reproducibility of the antibacterial effect and provide additional support for MIC determination based on progressive inhibition of bacterial growth rather than isolated observations. Variations among strains suggest a strain-dependent response to 3OMF exposure, although susceptibility was observed across all strains within the evaluated concentration range.

**Table 1:** Minimal inhibitory concentration of 3-*O*-methylfuricone (3OMF) and positive controls (amoxicillin, clarithromycin and metronidazole) against *H. pylori* ATCC 43504 and three clinical strains (PX256792, PX256793, and PX256794)

Sample	ATCC 43504	PX256792	PX256793	PX256794
3OMF	0.165 mM	0.660 mM	0.330 mM	0.660 mM
Amoxicillin	171.05 nM	684.18 nM	2736.76 nM	684.18 nM
Clarithromycin	20.89 nM	20.89 nM	20.89 nM	20.89 nM
Metronidazole	373.94 mM	46.74 mM	46.74 mM	46.74 mM

Targeted urease inhibitory activity was investigated to complement the results regarding inhibition of *H. pylori* by 3OMF. Weak urease inhibition by 3OMF was observed, even at the highest tested concentration (16.97% at 2.64 mM). Additionally, a checkerboard assay was performed to evaluate the interaction of 3OMF with clarithromycin, amoxicillin, and metronidazole. Interestingly, an additive effect was observed with clarithromycin (FICI = 0.75). In contrast, interactions with amoxicillin (FICI = 2.00) and metronidazole (FICI = 1.00) were classified as indifferent (Krzyżek et al. 2020).

Finally, the effects of 3OMF were evaluated in gastric adenocarcinoma (AGS, CRL-1739) and fibroblast (ATCC NCTC clone 929, CCL-1) cell lines. 3OMF exhibited selective cytotoxic activity against gastric adenocarcinoma cells, with IC_50_ values of 0.24 ± 0.03 mM, whereas an IC_50_ value of 0.57 ± 0.01 mM was observed for fibroblasts (SI = 2.37). The positive control cisplatin showed IC_50_ values of 37.96 ± 1.10 µM for gastric adenocarcinoma cells and 19.30 ± 0.87 µM for fibroblasts (SI = 0.51).

#### Molecular docking

Molecular docking simulations were performed to analyze the interactions between 3OMF and the following *H. pylori* target proteins: flagellar export chaperone FliS (PDB ID: 31QC), switch protein that regulates the rotation and switching of the flagellar motor FliM (PDB ID: 4GC8), replicative helicase DnaB (PDB ID: 4ZC0), β-clamp elongation factor crystallized with a DNA ligase peptide (PDB ID: 5FRQ), response regulator CheY1 co-crystallized with Mg²⁺ and BeF₃⁻ (PDB ID: 3H1E), glutamine synthetase (PDB ID: 5ZLI), and urease maturation protein UreE crystallized with Ni²⁺ (PDB ID: 3NY0).

**Fig. 4:**
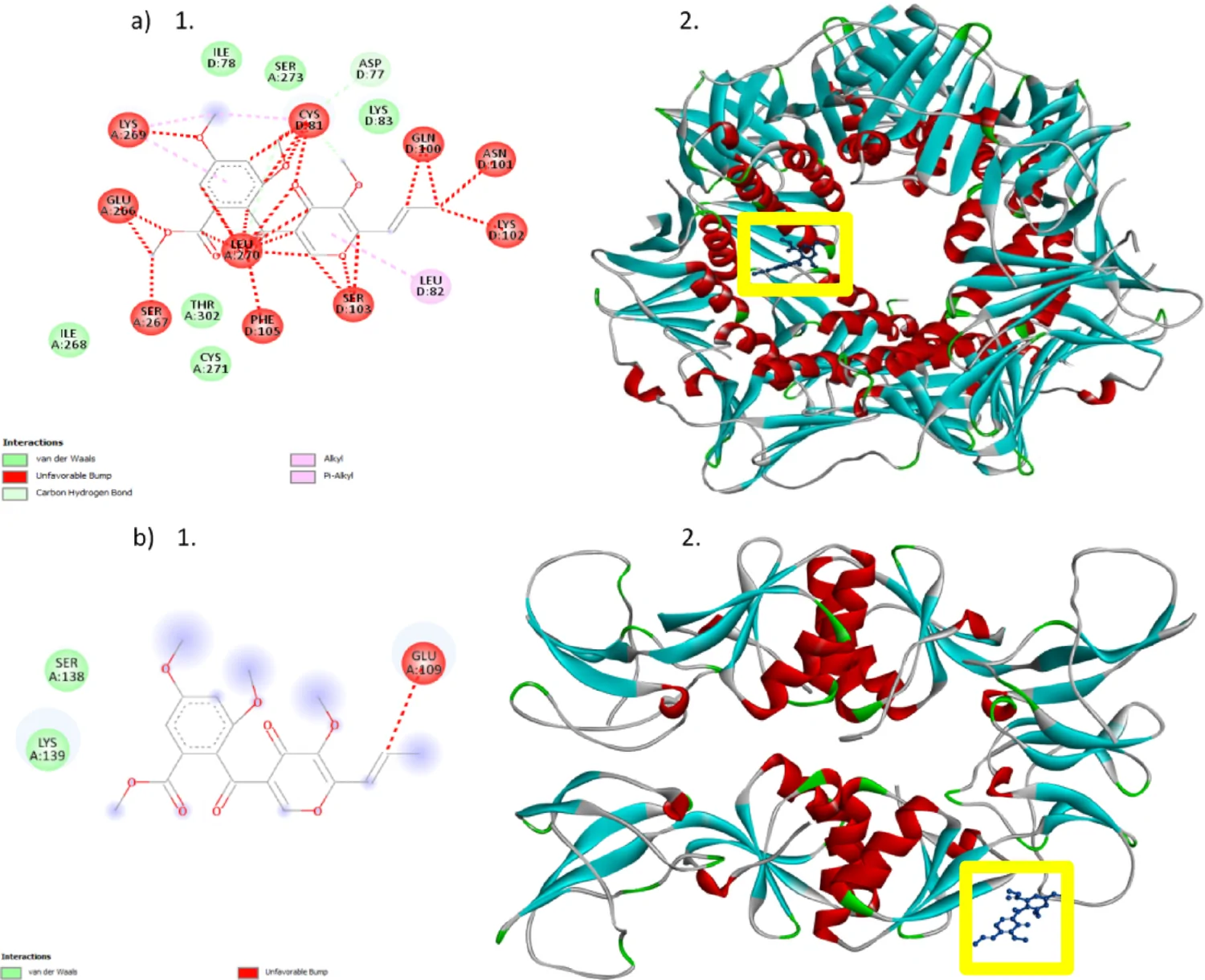
Intermolecular interactions between 3-*O*-methylfunicone and proteins **a** β-clamp and **b** urease; (1) The most likely intermolecular interactions are identified; (2) Three-dimensional structure of the ligand and protein (Red = α-helix, Cyan = β-sheet, Green/Gray = loops). The ligand is docked into the binding cavity of the proteins. The protein backbone is represented as a cartoon. The ligand (dark blue, highighlighted in yellow) (colour figure online)

The molecular docking was favorable to β-clamp and urease (Fig. 4). The results indicated binding affinity between 3OMF towards the β-clamp elongation factor and the urease maturation protein, with binding energies of − 5.56 [DN1] kcal/mol and − 4.57 kcal/mol, respectively. The docking results of β-clamp protein demonstrate that the ligand engages with the target protein through a diverse network of interactions, including *Pi*-Alkyl, *Pi*-Sigma, and carbon hydrogen bonding with residues that are also important for co-crystallized ligand stabilization, such as Phe243, Ile248, and Leu368. In contrast, UreE-3OMF complex was mainly stabilized by polar interactions between the carbonyl groups of the funicone groups and residues as His102 and His152, which are involved in endogenous nickel coordination (Fig. 4).

## Discussion

The isolation of metabolites with potent antibacterial activity from endophytic fungi represents a rational and promising strategy in natural product discovery. These microorganisms inhabit the endosphere of plants, where they coexist within complex microbial communities under conditions of intense ecological competition (Compant et al. 2021). Such an environment drives endophytic fungi to produce a diverse array of specialized metabolites, many of which function as chemical defenses against competing microorganisms. As a result, investigating endophytic fungi not only expands the chemical diversity available for drug discovery but also increases the likelihood of identifying novel molecules with unique mechanisms of action, which are urgently needed to combat the rise of antibiotic-resistant pathogens.

In this study, the endophytic fungus *T. pinophilus* J6, isolated from *E. umbellata* leaves, was investigated as a potential producer of anti-*H. pylori* natural products. Species of the fungal genus *Talaromyces* are widely recognized as important sources of funicone-like compounds, natural products that have attracted increasing attention due to their potential pharmaceutical applications. Among them, the 3OMF is a benzo-γ-pyrone derivative that displays antifungal activity (Nicoletti et al. 2015).

Changes in osmotic conditions induced by salt supplementation may act as environmental stressors, modulating fungal specialized metabolism by influencing the expression of biosynthetic gene clusters and triggering the production of natural products in filamentous fungi (Ariantari et al. 2019). The enhanced production of 3OMF observed under salt supplementation may therefore reflect a physiological adaptation to osmotic stress, although the molecular mechanisms responsible for this response remain to be elucidated. Additionally, microorganisms typically produce bioactive specialized metabolites during the late stages of growth, often reaching maximum levels during the late exponential or stationary phases, when nutrients become limited or cells are exposed to environmental stress. Optimizing cultivation time in this study enabled the identification of the period associated with the highest accumulation of 3OMF, suggesting that its biosynthesis is temporally regulated.

As part of the ongoing efforts to achieve pure natural products from endophytic cultures at sufficient yields for biological assays, the production of 3OMF by *T. pinophilus* J6 was optimized. Distinct culture media compositions (supplemented with inorganic salts) and incubation times were evaluated. UHPLC/ESI-Q-Orbitrap data (Fig. 2) were used to monitor the changes in the chemical profiles of the endophytic fungus grown in distinct conditions.

BPC data analysis showed that the main specialized metabolite detected in all culture conditions was the polyketide 3OMF (RT 23.83 min). Initially, the 3OMF was putatively identified in the crude extracts by manual curation of its MS1 and MS2 spectra, along with proposed fragmentation routes (Fig. 3). This analysis confirmed the presence of the target compound in all conditions. Comparisons between peak areas corresponding to 3OMF in the BPCs indicated that supplementation of PDA with sodium tartrate (PDA_ST) or ammonium sulfate (PDA_AS) enhanced 3OMF production by *T. pinophilus* J6. More specifically, cultivation in PDA_AS resulted in the highest 3OMF accumulation, approximately 10-fold higher than that observed in PDA_ST.

Subsequently, the time-course of 3OMF biosynthesis in PDA_AS medium was investigated to determine the optimal fermentation time for the highest yield of pure 3OMF. Time-course monitoring revealed that 3OMF production begins on the first day of cultivation and increases progressively, reaching its maximum level on the seventh day (Graph 1). This profile indicates continuous accumulation of the compound throughout the cultivation period, suggesting that its biosynthesis is sustained over time and may be associated with the late exponential or stationary phase of fungal growth.

In the present study, a one-variable-at-a-time (OVAT) approach was employed as a preliminary strategy to investigate factors affecting 3OMF biosynthesis by the endophyte strain J6. Initially, the culture medium composition was modified by adding inorganic salts, enabling identification of the optimal supplementation condition for enhancing 3OMF accumulation. Subsequently, cultivation time was optimized under the selected medium condition to determine the period associated with maximum metabolite production. Although this approach proved effective for the initial identification of cultivation parameters that influence 3OMF yield, it does not allow the assessment of interactions among variables. More comprehensive statistical optimization strategies could enable the simultaneous evaluation of multiple cultivation factors and their interactions, potentially leading to further improvements in 3OMF production.

Chromatographic isolation of the crude extract from scale-up of *T. pinophilus* J6 cultures in PDA_AS for 7 days yielded 3OMF as a pure compound, and its chemical structure was unequivocally identified by NMR and HRMS data analysis. Spectroscopic and spectrometric analyses ensured the compound’s purity and the reliability of its chemical characterization for downstream assays.

Initially recognized for their role in microbial antagonism, funicones and their analogs have attracted significant attention for their notable bioactivity, positioning them as candidates for drug discovery (Manzo and Ciavatta 2012). Over time, the antifungal and antiviral properties of 3OMF have been shown (Nicoletti et al. 2015; Santos et al. 2025), however, its antibacterial activity has not yet been comprehensively investigated, thus its inhibitory potential against *H. pylori* has not been reported so far.

*H. pylori* is a pathogenic bacterium that colonizes the human stomach and is strongly associated with chronic gastritis and gastric inflammation. Although many infections are asymptomatic, a significant proportion can progress to serious conditions, including gastric adenocarcinoma (de Martel et al. 2020). Standard treatment over the past two decades has relied on triple therapy combining clarithromycin, amoxicillin, and metronidazole with acid-suppressing agents. However, widespread use of these drugs has contributed to increasing antibiotic resistance, resulting in treatment failure rates exceeding 20% (Boyanova et al. 2023). In this context, natural products are gaining attention as alternatives against multidrug-resistant microorganisms, as they often exhibit biological activity and improved safety profiles. Several anti-*H. pylori* natural products from diverse plant and microbial sources have been reported for their role in the management of *H. pylori* infection (Padhi et al. 2025), including the polyketide armeniaspirol A, isolated from the culture of *Streptomyces armeniacu* that demonstrated antibiofilm activity (Jia et al. 2022).

In this study, the isolation and structural identification of the polyketide 3OMF established the chemical basis for its subsequent biological evaluation. Our findings demonstrated that 3OMF is a novel anti-*H. pylori* agent, which supports its further investigation as a potential drug lead for the treatment of *H. pylori* infection. Anti-*H. pylori* assays were carried out against one reference strain and three clinical isolates (Table 1), with MIC values ranging from 0.165 to 0.660 mM and bactericidal concentrations up to 1.32 mM. The clinical strains tested are resistant to amoxicillin, which may account for the reduced inhibitory activity of 3OMF against these isolates. Interestingly, 3OMF displayed greater antibacterial activity than metronidazole across all tested strains, as indicated by its markedly lower MIC values.

In checkerboard assays, 3OMF showed an additive effect with clarithromycin (FICI = 0.75), suggesting its potential as an adjuvant agent for the treatment of strains with reduced susceptibility to this antibiotic. In contrast, interactions with amoxicillin (FICI = 2.00) and metronidazole (FICI = 1.00) were classified as indifferent, indicating likely independent mechanisms of action.

*H. pylori* infection is recognized as a major cause of gastric cancer and represents the most common infection-associated malignancy (Baj et al. 2020). In this context, the effects of 3OMF were evaluated in gastric adenocarcinoma and fibroblast cell lines. Although 3OMF exhibited lower activity against the gastric adenocarcinoma cell line than cisplatin, its selectivity index (SI = 2.37) was more favorable than that displayed by cisplatin (SI = 0.51). This finding indicates that 3OMF was approximately twofold less toxic to non-tumoral cells than to gastric adenocarcinoma cells. Although compounds with higher inhibitory activity may be desirable, substances with SI values higher than 2.0 are generally considered to present acceptable safety profiles due to their potential for broader therapeutic windows (Badisa et al. 2006). The inhibitory activity of 3OMF against several cancer cell lines has been documented. Its proposed mechanism of action involves the activation of pro-apoptotic genes and the inhibition of cyclins. Moreover, the biological effect appears to depend on the methoxylated aryl group, which confers structural similarity to known antitumor agents such as combretastatin, podophyllotoxins, and chalcones (Nicoletti et al. 2009).

*H. pylori* survives in acidic environments, such as gastric and duodenal mucous, due to the production of urease, which converts urea into ammonia and thereby neutralizes gastric acidity (Carlini and Ligabue-Braun 2016). Studies have shown that urease-deficient mutants are unable to effectively colonize the gastric mucosa and consequently fail to induce gastritis (Debowski et al. 2017). Based on this evidence, urease inhibition was investigated to complement the results regarding inhibition of *H. pylori* by 3OMF. Weak urease inhibition by 3OMF was observed, even at the highest tested concentration (16.97% at 2.64 mM). The presence of the aromatic ring in 3OMF chemical structure may limit favorable interactions with polar residues in the urease active site, such as histidines and nickel-coordinated water molecules (Minkara et al. 2014). These findings are consistent with the molecular docking results for urease, which demonstrated weak binding affinity and distinct interaction profiles (Fig. 4).

To gain insight into the potential mechanism underlying the anti-*H. pylori* activity of 3OMF, molecular docking simulations were performed to investigate its interactions with the following proteins: flagellar export chaperone FliS, switch protein that regulate the rotation and switching of the flagellar motor FliM, replicative helicase DnaB, β-clamp elongation factor crystallized with a DNA ligase peptide, response regulator CheY1 crystallized with Mg²⁺ and BeF₃⁻, glutamine synthetase, and urease enzyme UreE crystallized with Ni²⁺. Although our investigations could cover different classes of proteins expressed by *H. pylori*, the docking results predicted favorable interactions only between 3OMF and the β-clamp and urease (Fig. 4).

The *in silico* results indicated that 3OMF exhibited moderate binding affinity toward the β-clamp elongation factor and the urease enzyme, with binding energies of − 5.56 kcal/mol and − 4.57 kcal/mol, respectively. The docking results of β-clamp protein demonstrate that the ligand engages with the target protein through a diverse network of interactions, including *Pi*-Alkyl, van der Waals, and carbon hydrogen bonding. Its ability to contact 12 residues across two protein chains and penetrate deeply into the subunit interface highlights its binding potential. In contrast, interactions with UreE involved only two amino acid residues, which is consistent with the less negative binding energy value. Therefore, β-clamp has more stable and well-distributed interactions within the binding site, suggesting greater stability of the ligand–protein complex.

To validate the docking protocol, a redocking procedure was performed for β-clamp using its corresponding reference ligand, resulting in a binding energy of − 6.4 kcal/mol. The similarity between the binding energy values obtained from docking and redocking supports the reliability of the simulations, indicating that the adopted model is suitable for predicting ligand–protein interactions in a reproducible manner. Although the molecular docking results suggested the possibility of interaction between 3OMF and the proposed targets, these findings should be interpreted as exploratory and hypothesis-generating, since docking analyses alone do not provide mechanistic confirmation and require further experimental and computational validation.

Inhibition of the β-clamp protein may affect the stability of the replication complex, impairing DNA polymerase activity and consequently limiting the proliferation of *H. pylori* (Pandey et al. 2016). The interaction with UreE suggests a possible interference with urease maturation, particularly due to the interactions predicted near the metal coordination sites of the enzyme. These findings provide a foundation for further optimization and position 3OMF as a promising scaffold for structure-based drug discovery. Although these interactions require experimental validation, the docking results suggest that 3OMF possesses structural features that enable interaction with essential bacterial proteins and may contribute to targeting virulence factors that allow bacterial survival in the gastric environment.

## Conclusion

This study demonstrates that culture optimization is an effective strategy to significantly enhance the production of 3-*O*-methylfunicone (3OMF) by the endophytic fungus *Talaromyces pinophilus* J6. Among the tested conditions, PDA supplemented with 45% (w/w) ammonium sulfate and a 7-day cultivation period provided the highest accumulation of the target metabolite, enabling its isolation as a pure compound and in sufficient amounts for comprehensive biological evaluation.

The isolated 3OMF showed antibacterial activity against *H. pylori*, including clinical strains, with bacteriostatic and bactericidal effects in the micromolar range. Although its direct urease inhibition was limited, the compound demonstrated an additive effect with clarithromycin, highlighting its possible application as an adjuvant molecule in combination therapies against resistant *H. pylori* infections. In addition, 3OMF displayed selective cytotoxicity toward gastric adenocarcinoma cells, with a favorable selectivity index compared with fibroblasts, reinforcing its potential pharmacological relevance in gastric disease-associated contexts.

Molecular docking analyses complemented the experimental findings by identifying β-clamp as a potential molecular target, suggesting that interference with bacterial DNA replication may contribute to the anti-*H. pylori* effect of 3OMF. Altogether, these findings expand the known bioactivity profile of 3OMF, establish an efficient platform for its production, and lay the groundwork for future structural modifications. Rather than proposing immediate therapeutic application, this work provides a foundational framework supporting the further investigation of 3OMF as a lead scaffold for the development of novel antimicrobial alternative therapies.

## Supplementary Information

Below is the link to the electronic supplementary material.


Supplementary Material 1


## Data Availability

No datasets were generated or analysed during the current study.
