# Supplementary material for: Enhancing 3-O-methylfunicone production by endophytic Talaromyces pinophilus J6 through culture optimization and its anti-Helicobacter pylori activity
Source: Arch Microbiol. 2026 Jul 9;208(9):474. doi: 10.1007/s00203-026-05028-9 (PMC13350209; doi:10.1007/s00203-026-05028-9)
Supplement: Supplementary file 1 — Supplementary Material 1 [file 203_2026_5028_MOESM1_ESM.docx]

**Supplementary Material**

**Enhancing 3-O-Methylfunicone Production by Endophytic *Talaromyces pinophilus* J6 Through Culture Optimization and Its Anti-*Helicobacter pylori* Activity**

Marcus V. A. Marques^1†^, Dalila N. Loose^2†^, Crislaine S. Lima^1^, Stéfane M. Q. Santos^1^, Cecília L. S. Pereira^1^, Gabriel S. Ramos^2^, Lorena C. Queiroz^2^, Rita C. R. Gonçalves^2*^, Eliane O. Silva^1*^

*^1^Department of Organic Chemistry, Institute of Chemistry, Universidade Federal da Bahia, Salvador 40170-115, Bahia, Brazil, elianeos@ufba.br*

*^2^Department of Pharmaceutical Sciences and Graduate Program in Pharmaceutical Sciences, Center of Health Sciences, Federal University of Espirito Santo, Vitoria, 29047-105, Espirito Santo, Brazil, rita.goncalves@ufes.br*

^†^ These authors contributed equally

*Corresponding authors:

elianeos@ufba.br (E.O. Silva)

Institute of Chemistry, Department of Organic Chemistry, Federal University of Bahia (UFBA), Barão de Jeremoabo 147, 40170-115 Salvador, Bahia, Brazil.

rita.goncalves@ufes.br (R.C.R Gonçalves)

Department of Pharmaceutical Sciences, Graduate Program in Pharmaceutical Sciences, Health Sciences Center, Federal University of Espírito Santo, 29047-105, Vitoria, Espirito Santo, Brazil.


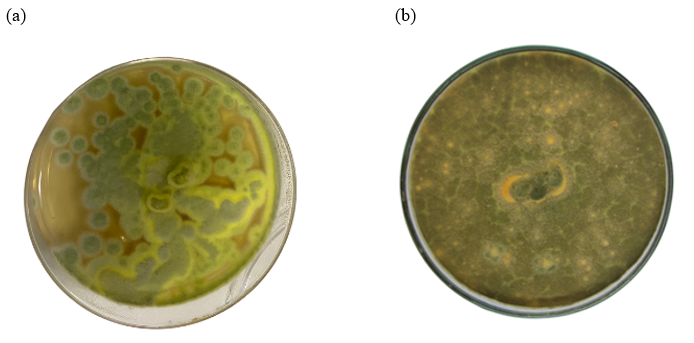


**Fig. S1.** Macroscopic morphology of 7-day cultures of *Talaromyces pinophilus* J6 grown on potato dextrose agar with (a) and without (b) ammonium sulfate


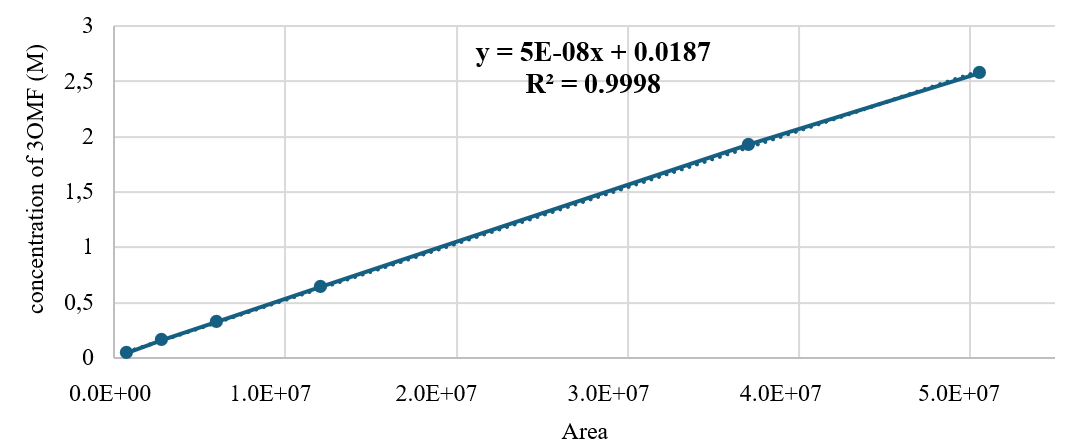


**Fig. S2.** Calibration curve of 3-*O*-methylfuricone (3OMF). Linear relationship between HPLC peak area and 3OMF concentration (M). The resulting regression equation was used to quantify 3OMF in fermentation samples.


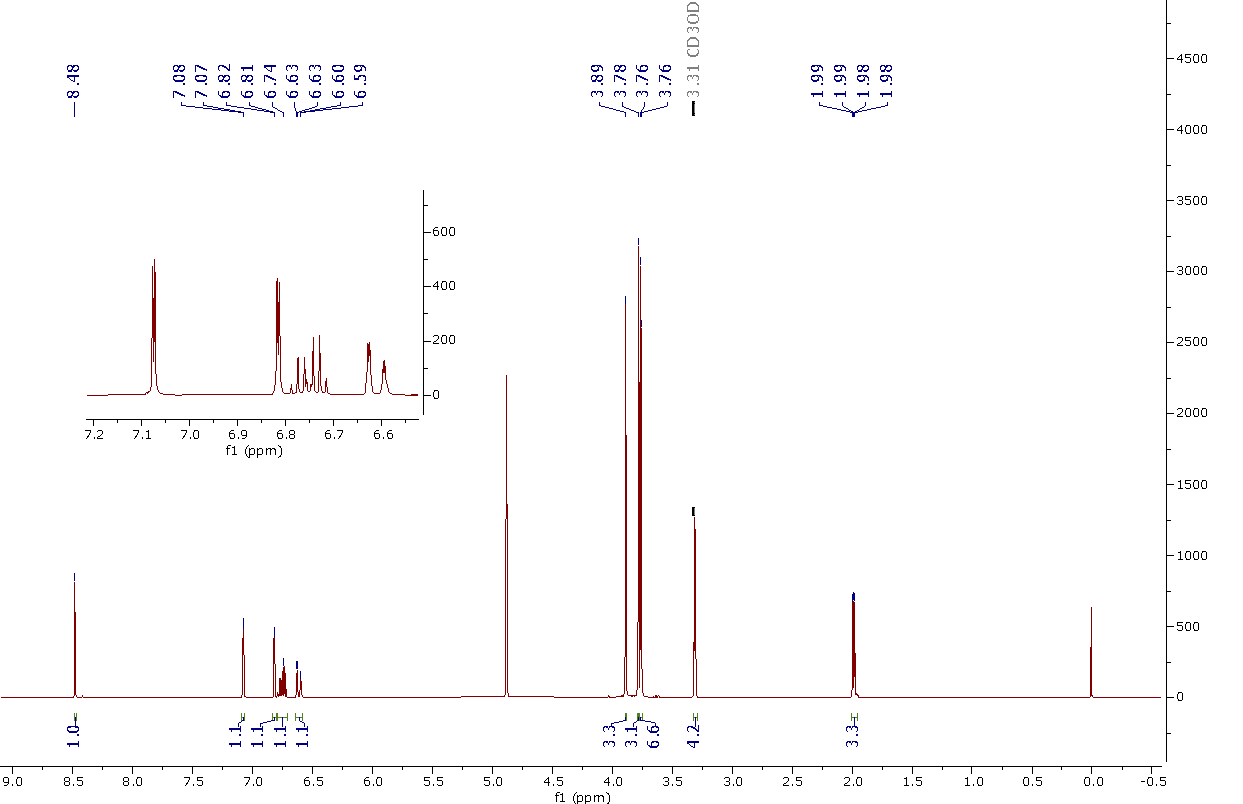


**Fig. S3.** 500 MHz ^1^H NMR spectrum of 3-*O*-methylfunicone registered in CD_3_OD


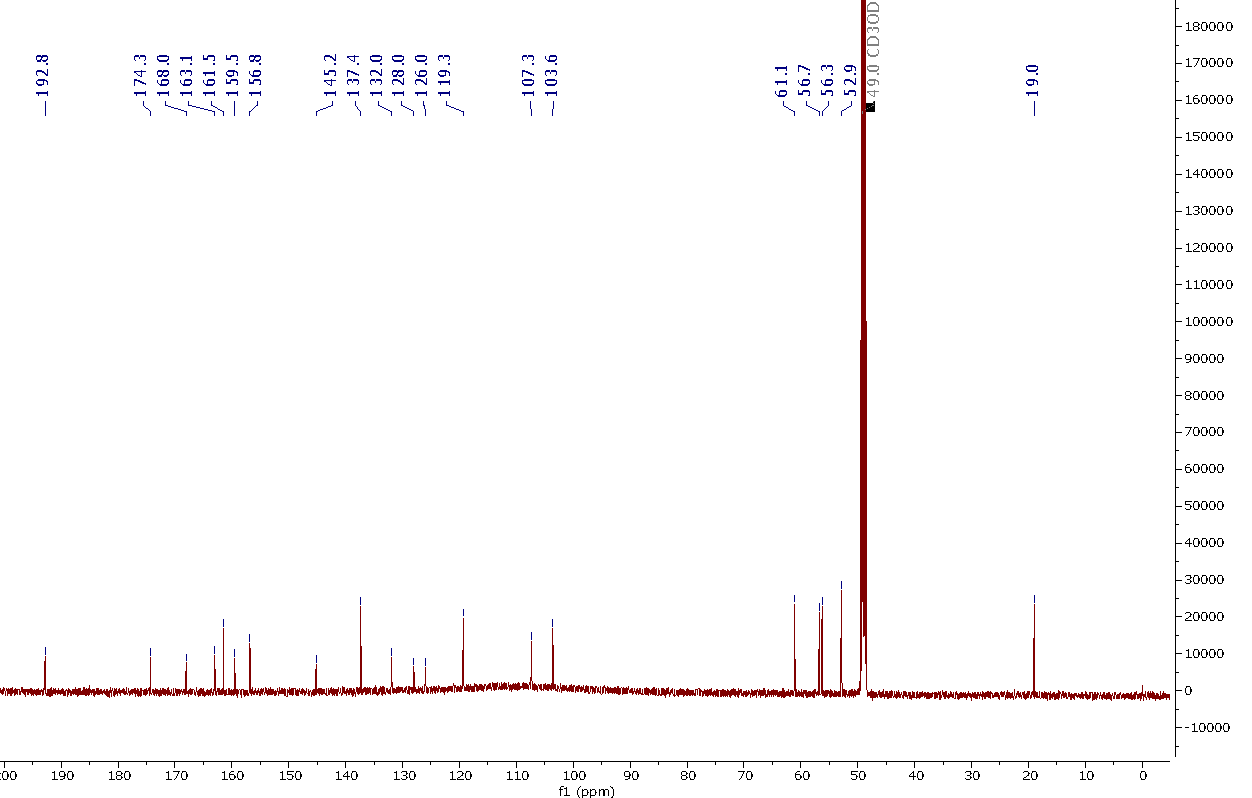


**Figure S4.** 125 MHz ^13^C spectrum of 3-*O*-methylfunicone registered in CD_3_OD


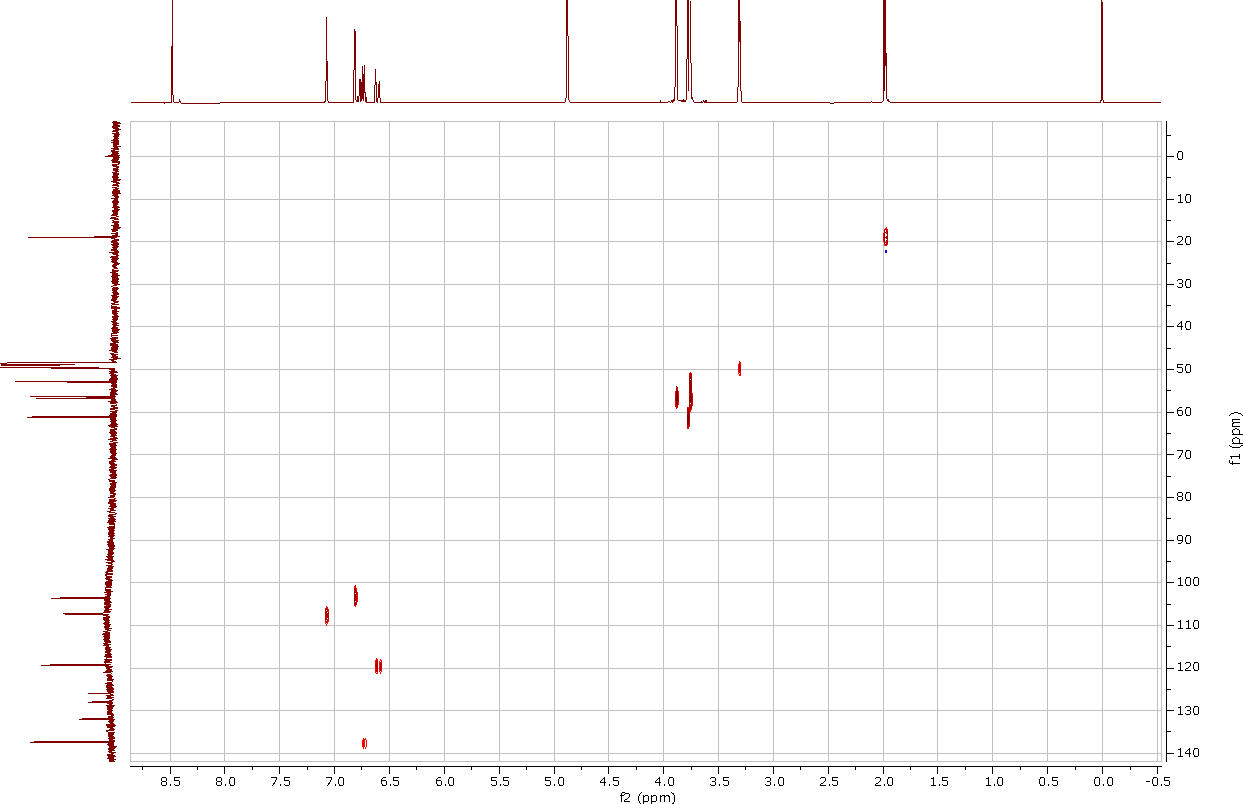


**Fig. S5.** Heteronuclear HSQC (500 MHz for ^1^H; 125 MHz for ^13^C) contour map of 3-*O*-methylfunicone registered in CD_3_OD


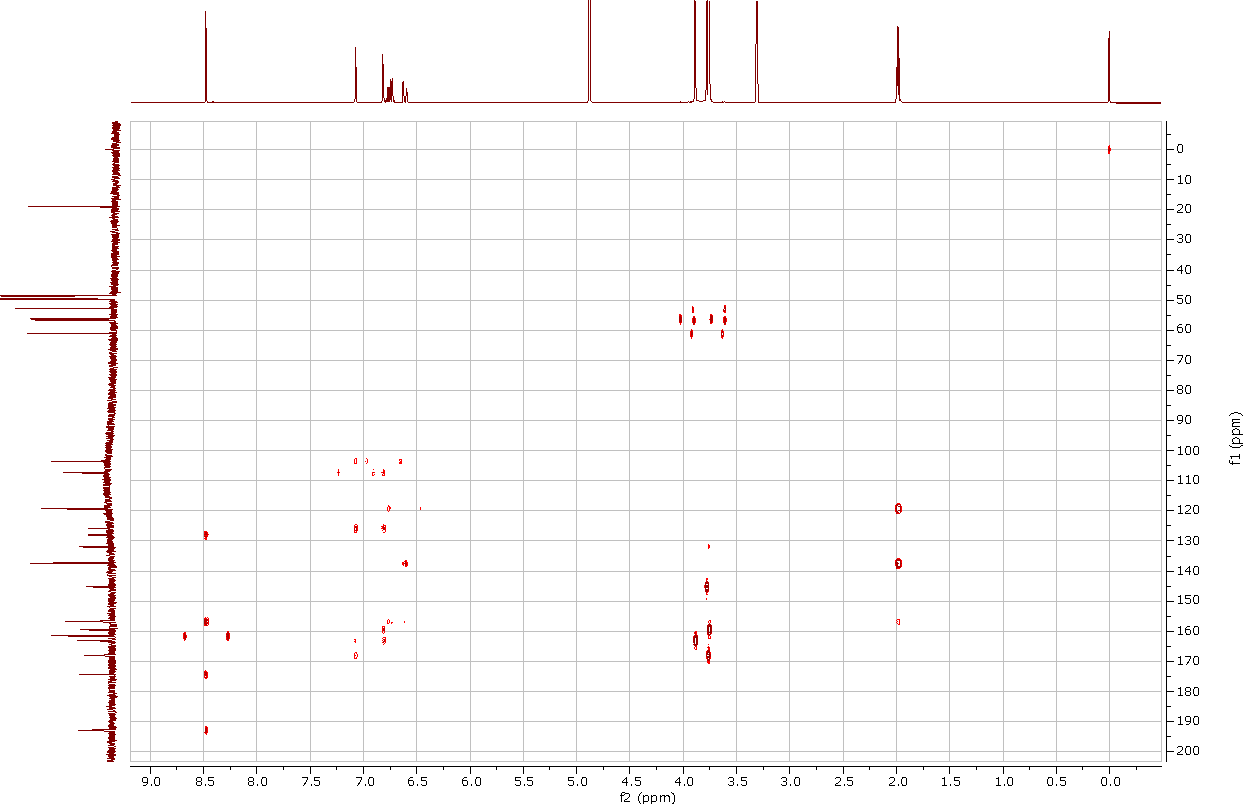


**Fig. S6.** Heteronuclear HMBC (500 MHz for ^1^H; 125 MHz for ^13^C) contour map of 3-*O*-methylfunicone registered in CD_3_OD

**
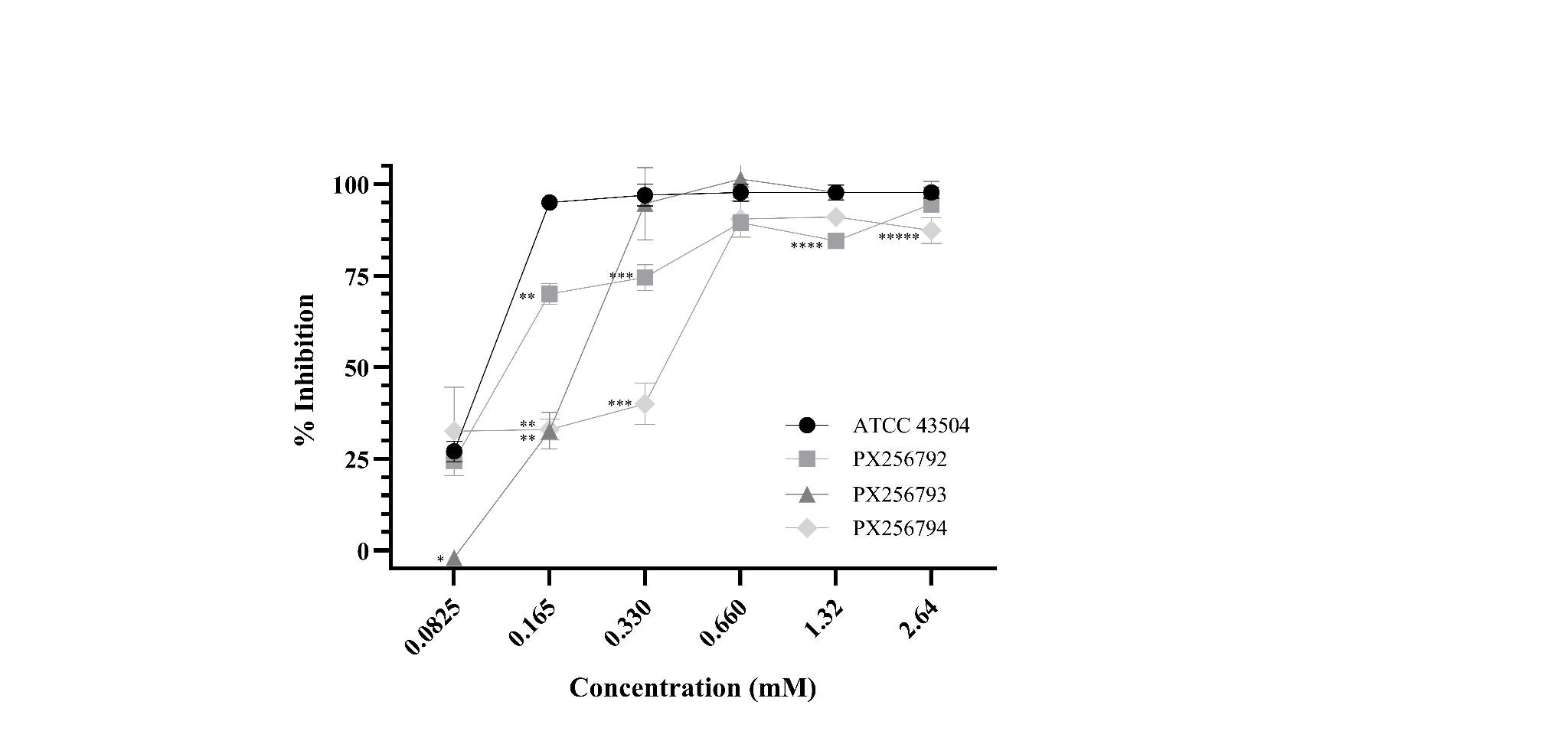
**

**Fig. S7.** Effect of 3-*O*-methylfuricone (3OMF) concentrations on *H. pylori* growth for ATTC 43504 and clinical strains (PX256792, PX256793 and PX256794). Results expressed as percentage of inhibition + standard deviation. Percentage inhibition values were analyzed by two-way ANOVA followed by Dunnett’s post-hoc test, with statistical significance set at p < 0.05.


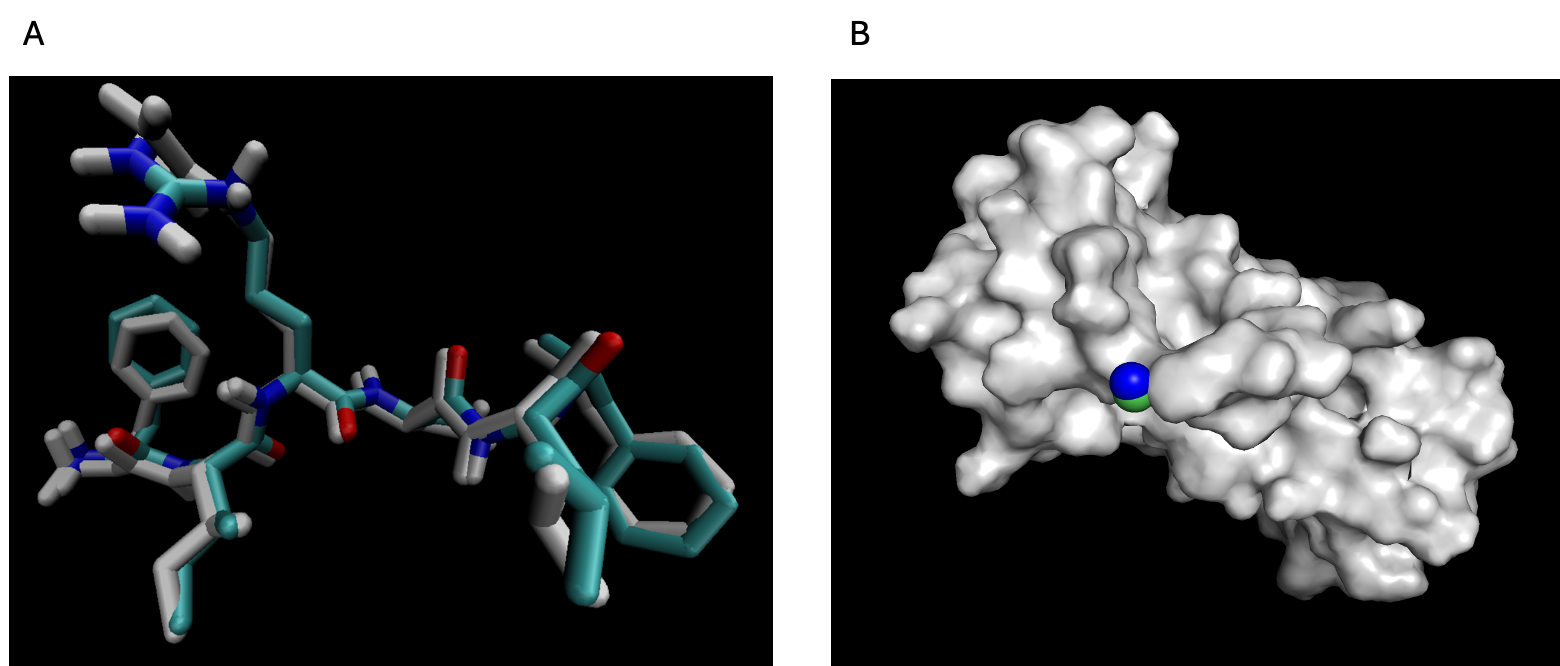


Fig. S8. Molecular docking protocol validation by redocking analysis. (A) Superposition between the crystallographic ligand (white) and the redocked pose (light blue) within the active site of *H. pylor*i β-clamp (PDB ID: 5FRQ). The redocking procedure yielded an RMSD value of 0.685 Å and binding energy of -6.8 kcal/mol. (B) Superposition between the crystallographic ligand (green) and the redocked pose (dark blue) within the active site of *H. pylori* UreE (PDB ID: 3NY0). The redocking procedure yielded an RMSD value of 0.959 Å and binding energy of -0.7 kcal/mol. RMSD values lower than 2.0 Å were considered indicative of satisfactory recovery of the crystallographic binding mode and were used to validate the docking protocol adopted in this study.
